# Efficacy and safety of artemether–lumefantrine as treatment for *Plasmodium falciparum* uncomplicated malaria in adult patients on efavirenz-based antiretroviral therapy in Zambia: an open label non-randomized interventional trial

**DOI:** 10.1186/s12936-019-2818-7

**Published:** 2019-05-24

**Authors:** Clifford G. Banda, Mike Chaponda, Mavuto Mukaka, Modest Mulenga, Sebastian Hachizovu, Jean B. Kabuya, Joyce Mulenga, Jay Sikalima, Linda Kalilani-Phiri, Dianne J. Terlouw, Saye H. Khoo, David G. Lalloo, Victor Mwapasa

**Affiliations:** 1 University of Malawi, College of Medicine, Blantyre, Malawi; 2 Malawi Liverpool Wellcome Trust Clinical Research Programme, Blantyre, Malawi; 3 Tropical Diseases Research Centre, Ndola, Zambia; 4 Oxford Centre for Tropical Medicine and Global Health, Oxford, UK; 5 Mahidol-Oxford Tropical Medicine Research Unit, Bangkok, Thailand; 6 Liverpool School of Tropical Medicine, Liverpool, UK; 7 University of Liverpool, Liverpool, UK; 8 Tropical and Infectious Diseases Unit, Royal Liverpool University Hospital, Liverpool, UK

**Keywords:** Human immunodeficiency virus, Anti-retroviral drugs, Artemether–lumefantrine, Malaria, Drug–drug interactions

## Abstract

**Background:**

HIV-infected individuals on antiretroviral therapy (ART) require treatment with artemisinin-based combination therapy (ACT) when infected with malaria. Artemether–lumefantrine (AL) is the most commonly used ACT for treatment of falciparum malaria in Africa but there is limited evidence on the safety and efficacy of AL in HIV-infected individuals on ART, among whom drug–drug interactions are expected. Day-42 adequate clinical and parasitological response (ACPR) and incidence of adverse events was assessed in HIV-infected individuals on efavirenz-based ART with uncomplicated falciparum malaria treated with AL.

**Methods:**

A prospective, open label, non-randomized, interventional clinical trial was conducted at St Paul’s Hospital in northern Zambia, involving 152 patients aged 15–65 years with uncomplicated falciparum malaria, who were on efavirenz-based ART. They received a 3-day directly observed standard treatment of AL and were followed up until day 63. Day-42 polymerase chain reaction (PCR)-corrected ACPRs (95% confidence interval [CI]) were calculated for the intention-to-treat population.

**Results:**

Enrolled patients had a baseline geometric mean (95% CI) parasite density of 1108 (841–1463) parasites/µL; 16.4% (25/152) of the participants had a recurrent malaria episode by day 42. However, PCR data was available for 17 out of the 25 patients who had malaria recurrence. Among all the 17 patients, PCR findings demonstrated malaria re-infection, making the PCR-adjusted day-42 ACPR 100% in the 144 patients who could be evaluated. Even when eight patients with missing PCR data were considered very conservatively as failures, the day-42 ACPR was over 94%. None of the participants, disease or treatment characteristics, including day-7 lumefantrine concentrations, predicted the risk of malaria recurrence by day 42. AL was well tolerated following administration. There were only two cases of grade 3 neutropaenia and one serious adverse event of lobar pneumonia, none of which was judged as probably related to intake of AL.

**Conclusions:**

AL was well tolerated and efficacious in treating uncomplicated falciparum malaria in HIV co-infected adults on efavirenz-based ART. However, a higher than anticipated proportion of participants experienced malaria re-infection, which highlights the need for additional malaria prevention measures in this sub-population after treatment with AL.

*Trial registration* Pan African Clinical Trials Registry (PACTR): PACTR201311000659400. Registered on 4 October 2013. https://pactr.samrc.ac.za/Search.aspx

**Electronic supplementary material:**

The online version of this article (10.1186/s12936-019-2818-7) contains supplementary material, which is available to authorized users.

## Background

Malaria and human immunodeficiency virus (HIV) infections co-exist in most parts of sub-Saharan Africa [1]. In these settings, antiretroviral therapy (ART) naïve HIV-infected (HIV+) individuals are more susceptible to falciparum malaria infection than HIV-negative individuals [2, 3]. The World Health Organization (WHO) recommends the use of artemisinin-based combination therapy (ACT) to treat falciparum malaria infections [4]. Artemether–lumefantrine (AL) is one of the most commonly used ACT in sub-Saharan Africa and is often co-administered with ART in malaria-HIV co-infected individuals.

First-line ART regimens in many parts of sub-Saharan Africa contain non-nucleoside reverse transcriptase inhibitors such as efavirenz. Efavirenz is metabolized by cytochrome (CYP) 450 enzymes, particularly CYP2B6 and to a lesser extent CYP3A4, which also metabolizes artemisinin-derivatives and lumefantrine, the longer-acting partner drug of AL [5, 6]. In addition, efavirenz auto induces its own metabolism [7] which may lower its plasma concentration and that of other CYP3A4 enzyme substrates, such as AL. Genetic variations in these enzymes are therefore likely to result in different levels of drug–drug interactions between AL and efavirenz. In pharmacokinetic studies, co-administration of efavirenz has been shown to be associated with lower lumefantrine exposure (nearly 50% reduction) than when administered alone [8–10]. In non-pregnant adults and children, lower lumefantrine concentrations have been associated with increased risk of recurrent malaria infections [11–14]. However, few studies have used the WHO-recommended protocol [15] to evaluate the efficacy of AL in HIV+ individuals on ART suffering from uncomplicated malaria. Hence, there are gaps in the evidence base that inform malaria treatment guidelines for HIV-infected individuals who are on ART.

A prospective, open label, non-randomized, interventional clinical trial was conducted to estimate the day-42 polymerase chain reaction (PCR)-corrected, adequate clinical and parasitological response (ACPR) and incidence of adverse events in HIV+ adult patients with parasitologically confirmed, uncomplicated clinical falciparum malaria who were on efavirenz-based ART. Additionally, factors associated with malaria recurrence by day 42 of follow up, including day-7 plasma lumefantrine concentrations, were also assessed.

## Methods

### Study site and study population

The study was conducted from October 2014 to June 2015 at St Paul’s Hospital in Nchelenge district in the northern province of Zambia, which borders the Democratic Republic of Congo. The district had an estimated population of 147,127 according to the 2010 population census [16]. This is a setting of moderate-high transmission of malaria [17] and high HIV prevalence of 12.0% [18].

During the study period, the criteria for initiating ART in Zambia was WHO HIV disease stages 3 or 4, CD4 cell count < 350, pregnancy or lactation. First-line ART regimen comprised of tenofovir/lamivudine/efavirenz. AL was the first-line treatment for uncomplicated malaria.

### Study design and clinical procedures

This was a single arm interventional study (registration number: PACTR201311000659400). HIV-infected patients who had been on 600 mg efavirenz-based ART for at least 24 weeks and were suspected to have malaria were pre-screened through history taking and clinical examination to determine their eligibility for the study. The study inclusion criteria were as follows: age ≥ 15 to ≤ 65 years; weight ≥ 35 kg; documented fever (axillary ≥ 37.5 °C) or history of fever 24 h prior to enrolment; smear positive *pf* malaria mono-infection with asexual malaria parasite densities < 200,000/µL; ability to swallow oral medications and willingness and ability to comply with scheduled visits, supervised treatment administration, laboratory tests, and other study procedures. The following were the exclusion criteria: severe malaria as per WHO criteria [19]; mixed infection with another *Plasmodium* species; haemoglobin (Hb) concentration < 7 g/dL; severe sickle cell disease or sickle-haemoglobin C anaemia; evidence of pregnancy or lactation; use of any anti-malarial treatment or drug with anti-malarial activity within the past 1 month, except cotrimoxazole; history of AL hypersensitivity reactions; gastrointestinal diseases that could alter gut absorption or motility; history of splenectomy; history of epilepsy or convulsions; pre-existing clinically-significant cardiac, liver, renal, neurological or psychiatric abnormalities; alternative clinical cause of fever other than malaria and participation in any investigational drug study in the past 30 days.

Finger-prick blood samples were taken from those who satisfied the preliminary eligibility criteria and tested for malaria using rapid diagnostic test (RDT) (SD BIOLINE Malaria Ag P.f/Pan test produced by Alere) and for haemoglobin concentration using Hemocue Haemoglobinometer. Thick blood smear microscopy examinations were performed on patients with RDT-positive malaria. Clinical examinations were performed in those with confirmed malaria parasitaemia. Consenting participants were enrolled and scheduled for a 3-day hospital admission (treatment period) and then followed up until day 63.

Enrolled study participants received a directly observed, standard 3-day course of AL (Coartem^®^, Novartis) administered twice a day with milk as follows: 4 tablets of AL, each containing 20 mg/120 mg of artemether/lumefantrine administered at 0 h (treatment day 0), 8 (± 4) h after the first dose, then at 24 (± 4) and 36 (± 4) h after dose (treatment day 1) as well as 48 (± 4) and 60 (± 4) h after the first dose (treatment day 2). A repeat dose of AL was administered to participants who vomited within 30 min. Participants with persistent vomiting were withdrawn from the study and referred appropriately. Paracetamol ≤ 1 g was given concomitantly with the first dose of AL to relieve malaria symptoms. The paracetamol doses were repeated at intervals of 6 h, if clinically indicated. Participants’ vital signs were measured at 6-hourly intervals and adverse events were monitored. A 12-Lead electrocardiogram (ECG) was performed before the first dose of AL and within 2 h after administration of the third dose AL. Any patient with Fridericia corrected QT (QTcF) interval of ≥ 450 ms or QTc increase of > 60 ms from the baseline underwent follow-up ECGs until resolution of the abnormality. Participants were discharged at least 24 h after taking the third (last) dose of AL (post-treatment day 3) and advised to come for follow-up visits on post-treatment days 7 (± 1), 14 (± 1), 21 (± 2), 28 (± 2), 35 (± 2), 42 (± 2) and 63 (± 2). Participants were encouraged to return to the health facility any time they felt unwell (unscheduled visits). All adverse events were graded using the DAIDS criteria [20]. Adverse events with onset or increased severity after the first dose of AL were counted as treatment-emergent adverse events (TEAEs). During follow-up visits, participants’ time and any incurred expenses when attending the study clinic were appropriately compensated, as approved by the ethics committees.

### Laboratory procedures

During the admission period, thick blood slides were collected pre-dosing and at 6-hourly intervals until after obtaining two consecutive malaria negative smears. The slides were also collected at scheduled and unscheduled follow-up visits. The slides were Giemsa-stained and read by an experienced microscopist using standard protocols [21]. For quality control, all slides were re-read by a second microscopist; a third microscopist settled any discrepant readings. Dry blood spot (DBS) samples were collected on filter paper (Whatman 3MM^®^) at baseline and during recurrent malaria episodes. Parasite DNA was extracted from the DBS samples, amplified using polymerase chain reaction (PCR) and genotyped for merozoite specific protein (MSP) 1 and 2 to distinguish malaria recrudescence from re-infection, using methods previously described [22]. Samples that did not produce results were classified as indeterminate.

Venous blood samples were collected on days 0, 3, 28, 42, and 63 for biochemistry tests using a Beckman CX5^**®**^ Chemistry analyzer, on days 0, 3, 7, 28, 42, and 63 for haematological tests using a Beckman Coulter^**®**^ HMX Analyzer and on days 0, 28 and 63 for CD4 cell count measurement using a BD FACSCount™ machine. Plasma samples collected on days 0, 28 and 63 were stored for future HIV viral load assays. Additionally, sparse plasma samples were collected to quantify lumefantrine concentration during the follow-up period, these findings will be reported elsewhere.

On day 7, blood samples for lumefantrine PK assays were collected in heparin tubes. Immediately after collection, the blood samples were spun in a refrigerated centrifuge, and the separated plasma samples were temporarily frozen in liquid nitrogen before being transferred to a − 80 °C freezer until PK analyses. The plasma samples were analysed for lumefantrine levels at the Malawi-Liverpool-Wellcome Trust Clinical Research Programme in Blantyre, Malawi, using a validated high-performance liquid chromatography (HPLC)-UV assay adopted and transferred to Malawi from the Liverpool School of Tropical Medicine. The PK laboratory in Blantyre participated in WorldWide Antimalarial Resistance Network (WWARN) external quality assurance programme [23]. Briefly, lumefantrine and the internal standard (IS) (halofantrine) were recovered from plasma using a single protein precipitation step with acetonitrile and acetic acid (99:1). The supernatant was then evaporated to dryness in a vacuum concentrator at 25 °C. The dried extract was redissolved in the reconstitution solvent methanol-0.01 M hydrochloric acid (70:30), and 75 μL was injected into the chromatograph (Agilent 1100). Quantitation of the drugs was achieved by reverse-phase HPLC. The optimum detection wavelength for each drug was 335 nm. The lower limit of quantification (LLOQ) of the HPLC–UV assay was 50 ng/mL for lumefantrine, with a percent coefficient of variation of < 10. An extracted plasma pharmacokinetic sample from a participant was run in a batch comprising. Each batch run included a blank plasma extract, two sets of 8-concentration-level calibration standards, and quality controls (QCs) at three concentration levels: low, medium and high (0.05, 10 and 15 μg/mL). For the batch assay to pass, the measured concentrations of at least 67% of the QC samples had to be within ± 20% of their nominal value, and at least one QC had to be acceptable at the LLOQ. The mean interassay precision values for low, medium, and high QCs were 6.6, 8.8 and 9.2%, respectively. In addition, 75% of each calibration curve’s concentrations had to lie within ± 20 and ± 15% of the nominal concentration at the LLOQ or all other concentrations, respectively.

### Study endpoints

The primary study endpoint was proportion of patients with PCR-corrected day-42 ACPR, defined as patients who at day 42 did not have parasitaemia with identical falciparum malaria PCR markers (merozoite specific protein 1 and 2) to baseline, irrespective of axillary temperature, and who had not previously met any of the criteria of early treatment failure (ETF), late clinical failure (LCF) or late parasitological failure (LPF). Standard WHO definitions of ETF and LCF were used [15].

The other primary study endpoints were grade 3 or 4 TEAEs including cases of Fridericia corrected QT (QTcF) interval prolongation [24] and serious adverse events (SAEs) as per standard definitions [25]. Local study physicians determined the relationships between AL and the adverse events (AEs). A Data Safety and Monitoring Board (DSMB) reviewed the AEs and assessed the validity of study physicians’ decisions.

Secondary efficacy endpoints included PCR-uncorrected day-42 ACPR, time for parasite to decline by 50% (PC_50_) and 90% (PC_90_), fever clearance time, and day-7 lumefantrine concentrations in relation to day-42 ACPR. Additional exploratory analyses focused on identifying any potential predictors of not achieving ACPR (recrudescence) or acquiring re-infection by day 42. Secondary safety endpoints were trends in haemoglobin concentrations and CD4 cell counts from baseline to day 28.

### Sample size

Sample size calculation was based on estimates of total treatment failure rate (TTFR). The 42-day PCR-corrected TTFR was estimated to be ≤ 10% [26]. A precision of 5%, around this point estimate, which resulted in the upper limit of the 95% Wald binomial confidence interval to be 15%, was allowed. Using the formula for estimating a single study population sample size [27], the effective sample size was estimated at 138. The final sample size was 163, after adjusting for an anticipated loss-to-follow-up rate of 15%. Sample size calculations and subsequent statistical analyses were performed in STATA 13.1.

### Statistical analyses

For the primary endpoint, three analysis populations were used. Firstly, the intention-to-treat (ITT) population included patients who received at least 1 dose of study medication. Secondly, the per-protocol (PP) population included all participants who received a full course of AL, had data available for the primary endpoint at day 42, and who adhered to the follow-up visit schedule. Thirdly, the safety population included all patients who received any amount of study medication and had at least 1 assessment after dosing. ACPR plus 95% CI in PP and ITT populations were reported. The primary analyses was based on the ITT population while the PP populations assessed the robustness of assumptions that are made in the ITT approach. In the ITT approach, secondary sensitivity analyses were performed by firstly considering all participants with missing PCR data at day 42 as having parasitological failure and then considering the same participants as having treatment success.

Statistical analyses for secondary ACPR endpoints were similar to the primary endpoints. In addition, Kaplan–Meier (KM) survival plots were used to summarize the time to PCR-corrected and uncorrected treatment failure in the ITT population. Parameters assessing post-treatment parasite clearance (PC_50_ and PC_90_) were estimated using the WWARN parasite clearance estimator (WWARN PCE), as described elsewhere [28].

Descriptive statistics were computed for baseline variables. Wilcoxon rank-sum/Mann–Whitney U test was used to compare distributions of the day-7 lumefantrine concentrations in those who had and did not have malaria recurrence by day 42. Lumefantrine concentrations below the lower limit of quantification (< 50 ng/mL) were imputed to half the lower limit of quantification (25 ng/mL). Additionally, Wilcoxon matched paired signed-rank test was used to compare baseline and day-28 CD4 cell and haemoglobin values. Cox univariate and multivariate regression models were used to determine potential predictors of malaria recurrence by day 42 and to compute hazard ratios in the ITT population. In the univariate analysis, potential predictors such as age, gender, baseline CD4 cell count, malaria parasite density were determined as significant if they had a p-value of < 0.05. Significant variables in the univariate models were then fitted in a multivariate model and covariates that had a p-value of < 0.05 in the multivariable model were considered independent predictors of malaria recurrence.

## Results

### Study profile

**Fig. 1:**
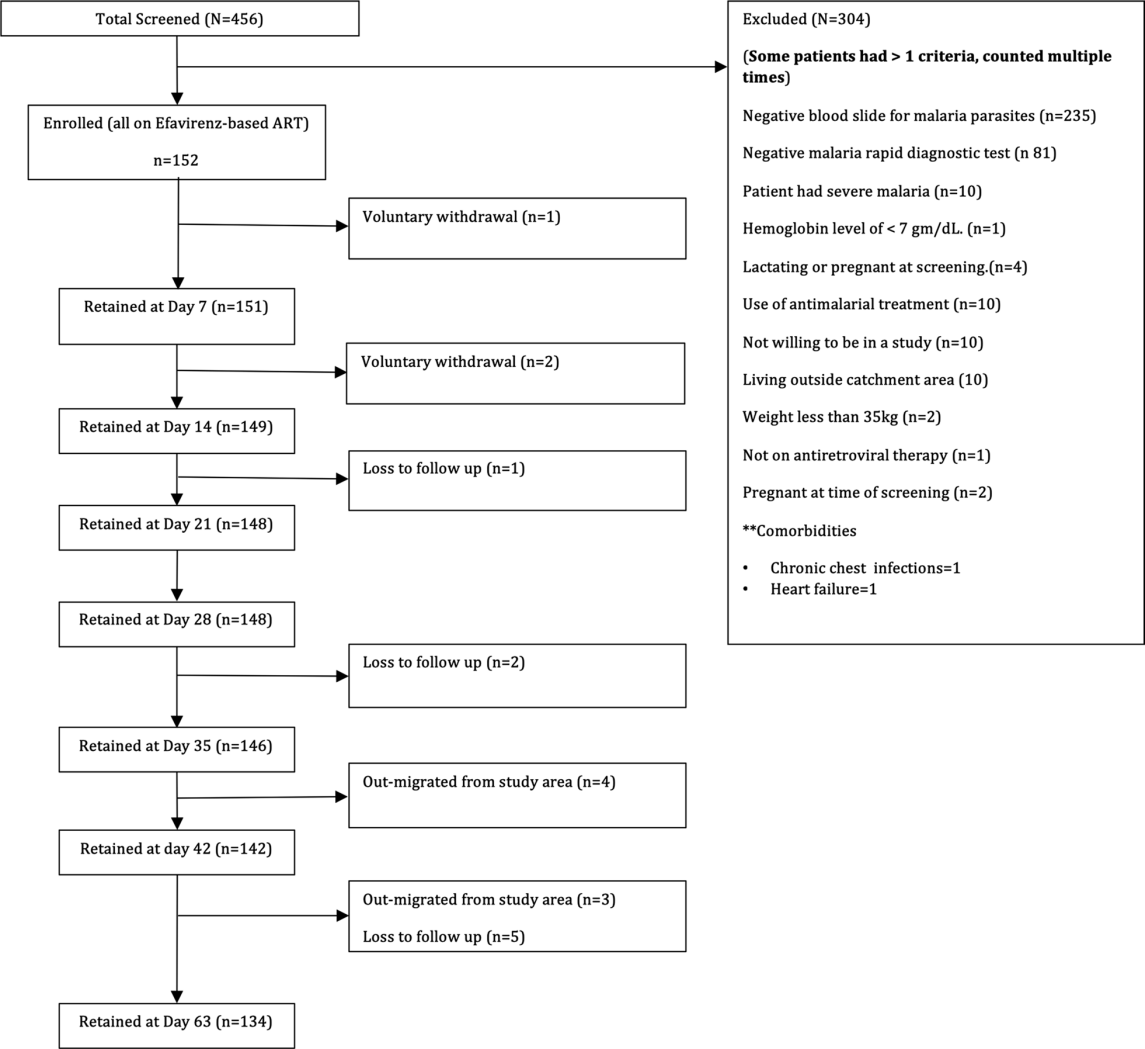
Trial profile and flow chart of participants. Trial profile and flow chart of malaria-HIV co-infected participants enrolled into in Zambia who were on efavirenz-based antiretroviral therapy and were treated for malaria with artemether–lumefantrine

As shown in Fig. 1, 456 patients presenting with symptoms suggestive of malaria at St Paul’s Hospital were screened for trial eligibility. Of these, 152 with positive falciparum malaria blood met the eligibility criteria and were enrolled in the trial. Thereafter, 6.6% (10/152) of the participants were lost to follow-up, withdrew consent or migrated from study area by day 42.

### Baseline characteristics

The baseline characteristics of enrolled study participants are summarized in Table 1. The median duration on ART was 2 years and almost two-thirds of the participants had a normal body mass index. Although over 80% of the participants reported fever at presentation, only a small proportion (7.9% [12/152]) were clinically febrile. Generally, the study population had a low median parasite density with only one-third having baseline parasite density exceeding 2000 per μL. Nearly 45% of the participants had CD4 cell count < 350 cell/cu mm. Only 34.9% (53/152) reported taking cotrimoxazole prophylaxis.

**Table 1:** Baseline characteristics of enrolled participants

Variable	Artemether–lumefantrine + efavirenz-based ART (N = 152)
Age in years, median (IQR)	40.4 (34–46.1)
Female (%)	101 (66.5)
Body mass index, kg/sq m, median (IQR)	19.5 (18.3–21.4)
WHO BMI classification, n (%)	
Underweight (< 18.5)	45 (29.6)
Normal (18.5–24.9)	99 (65.1)
Overweight (25.0–29.9)	5 (3.3)
Obese (> 30)	3 (2.0)
Duration on ART in months, median (IQR)	24 (10.5–48)
Presenting symptoms, n (%)	
Fever	124 (81.6)
Headache	119 (78.3)
Fatigue	47 (30.9)
Nausea	14 (9.2)
Axillary temperature at enrolment, median (IQR)	36.4 (36.0–36.7)
Febrile, n (%)	12 (7.9)
Geometric mean parasite density, 95% CI	1108 (841–1463)
Parasite density > 2000 parasites/µL, n (%)	53 (34.9)
Parasite density > 10,000 parasites/µL, n (%)	19 (12.5)
Pre-treatment haemoglobin concentration, g/dL, median (IQR)	11.3 (10.5–12.4)
Pre-treatment CD4 cell count, median (IQR)	376 (248–511)
CD4 cell count < 350, n (%)	67 (44.1)
Median (IQR) QTcF interval (ms)	393 (358–413)
Current use of cotrimoxazole prophylaxis, % (n)	53 (34.9)

### Treatment dosage and tolerability

The daily median (range) dosages of artemether and lumefantrine administered to participants were 3.2 mg/kg (2.3–4.6) and 19.2 mg/kg (13.5–27.4), respectively. The dosages were well tolerated: only one participant vomited following intake of AL on first day of treatment and was re-dosed. No participant was withdrawn from the study due to persistent vomiting.

### Treatment efficacy

As shown in Table 2, there were no cases of ETF or LCF. There were 25 cases of late parasitological failure by day 42 of follow-up (3 on day 28, 9 on day 35 and 13 on day 42). Seventeen of the 25 cases of late parasitological failure had parasite genotyping results available. All 17 cases were confirmed as reinfections using PCR.

**Table 2:** Efficacy outcomes, treatment success rates and sensitivity analyses by day 42 among the enrolled participants

Variable	Artemether lumefantrine + efavirenz-based ART N = 152
Early treatment failure-no. (%)	0
Late clinical failure-no. (%)	0
Late parasitological failure-no. (%)	25 (16.4)
Recrudescence	0
Reinfection	17 (11.2)
PCR indeterminate or sample unavailable	8 (5.2)
Treatment success rate by day 42	
PCR-unadjusted treatment success rate	
Intention-to-treat analysis	
Number of patients	152
Rate-% (95% CI)	83.6 (76.7–88.7)
Per-protocol analysis	
Number of patients	142
Rate-% (95% CI)	82.4 (75.1–87.9)
PCR-adjusted success rate excluding indeterminate or unavailable PCR samples	
Intention-to-treat analysis	
Number of patients	144
Rate-% (95% CI)	100
Per-protocol analysis	
Number of patients	134
Rate-% (95% CI)	100
Sensitivity analyses of treatment success rate by day 42	
PCR-adjusted success rate (scenario 1^a^)	
Intention-to-treat analysis	
Number of patients	152
Rate-% (95% CI)	100
Per-protocol analysis	
Number of patients	142
Rate-% (95% CI)	100
PCR-adjusted success rate (scenario 2^b^)	
Intention-to-treat analysis	
Number of patients	152
Rate-% (95% CI)	94.7 (89.8–97.4)
Per-protocol analysis	
Number of patients	142
Rate-% (95% CI)	94.4 (89.1–97.2)
Treatment success rate by day 42 according to use of cotrimoxazole^c^	
On cotrimoxazole prophylaxis in the intention-to treat analysis	
Number of patients	53
PCR-adjusted rate-% (95% CI)	100
Not on cotrimoxazole prophylaxis in the intention-to-treat analysis	
Number of patients	88
PCR-adjusted rate-% (95% CI)	100

*ART* antiretroviral therapy

^a^Scenario 1: indeterminate or unavailable PCR samples (as well as loss to follow-up in intention-to-treat population) treated as treatment success

^b^Scenario 2: indeterminate or unavailable PCR samples (as well as loss to follow-up in intention-to-treat population) treated as treatment failures

^c^Excluding 8 patients with indeterminate or unavailable PCR samples and 3 patients with missing information on cotrimoxazole use

In the analysis of complete case records, excluding the 8 participants with missing PCR sample by day 42, the PCR-adjusted day-42 ACPR was 100% in both the ITT and PP populations. Additional file 1 illustrates the adjusted estimate of the proportion that experienced treatment success by day 42 in the ITT population.

The PCR-unadjusted day-42 ACPR, in the ITT population was 83.6% (95% CI 76.7–88.7%) and 82.4% (95% CI 75.1–87.9%) in the PP population (Table 2). Additional file 2 shows the unadjusted estimate of the proportion that experienced treatment failure by day 42 in the ITT population. The majority of the treatment failures occurred between days 35 and 42 (9 and 13 cases, respectively).

Sensitivity analyses performed for patients with unavailable malaria parasitological outcomes (due to lost to follow-up, missed day 42 visit or missing thick smear slides) showed that when cases with missing day-42 PCR samples were treated as re-infections (scenario 1 in Table 2), the PCR-adjusted ACPR was 100%, as in the primary analysis. If cases with missing PCR samples were treated as recrudescent infections (scenario 2 in Table 2), the PCR-adjusted day-42 ACPR was still well above 90%.

### Parasite clearance time

Parasite clearance parameters were calculated in 57 and 54 participants in the ITT and PP populations, respectively, who had detectable parasitaemia at two or more post-dosing time points to allow determination of parasite clearance slope. The median (range) PC_50_ and the median (range) PC_90_ were 5.7 (0.3–25.8) h and 13.8 (2.9–32.9) h, respectively, in the ITT population and were 6.0 (0.3–24.4) h and 13.1 (2.9–30.9) h, respectively, in the PP population.

### Fever clearance

At baseline, 7.9% (12/152) of the participants were febrile (axillary temperature ≥ 37.5 °C). The median fever clearance time (IQR) was 6 (6–12) h. None of the participants was febrile on days 1 and 2 of treatment.

### Day-7 plasma lumefantrine concentrations and other predictors of malaria recurrence by day 42

On day 7 of follow-up, blood samples were available from 121 participants for quantification of lumefantrine concentrations. Among these, 36 participants had lumefantrine concentrations below the LLOQ (< 50 ng/mL). Since the proportion of participants with values below LLOQ was greater than 10%, the values below the LLOQ were handled in two ways as previously documented [29, 30]; in the first method only values above the LLOQ (n = 85) were used for analyses and in the second method, which was conducted as sensitivity analysis, values below LLOQ (n = 36) were included in the analyses after imputing the values to half the LLOQ (25 ng/mL).

**Fig. 2:**
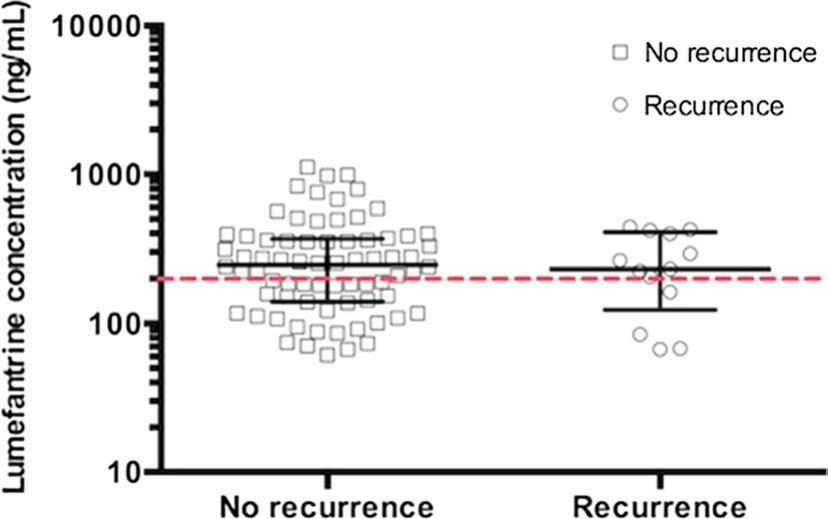
Day-7 plasma lumefantrine concentration among malaria-HIV-co-infected patients who had quantifiable concentrations above the lower limit of quantification (n = 85). Day-7 plasma lumefantrine concentration in malaria-HIV-co-infected patients who experienced malaria recurrence day 42 (n = 13) and those who did not experience recurrence day 42 (n = 72) in the intention-to-treat population. Individuals with unquantifiable or missing day-7 lumefantrine concentrations (n = 30), a participant who withdrew consent before day 7 and individuals with lumefantrine values below the lower limit of quantification [< 50 ng/mL] (n = 36) are excluded from the plot. Lumefantrine concentrations are given in ng/mL on a logarithmic scale. Red line is equivalent to 200 ng/mL. Black line through the dot indicates median and the bar represents the interquartile range

In those with day-7 lumefantrine values above the LLOQ, the median (IQR) lumefantrine concentrations was 240 [143.2–370] ng/mL. As shown in Fig. 2, the median (IQR) lumefantrine concentrations were not significantly different between participants who did not have malaria recurrence (246.8 [141.4–365.1] ng/mL) and those who had malaria recurrence by day 42 (230.8 [162.5–398.5] ng/mL, p = 0.779). In addition, the proportion [95% CI] that attained day-7 plasma lumefantrine concentrations ≥ 200 ng/mL was not significantly different between those who experienced (69.2% [95% CI 36.5–89.8]) and did not experience malaria recurrence by day 42 (58.3% [95% CI 46.4–69.3]; p = 0.460). None of the 36 participants who had day 7 lumefantrine concentrations below the LLOQ experienced malaria recurrence by day 42. Even after imputing data for these participants with lumefantrine values below the LLOQ, there was no difference in median day-7 lumefantrine concentration and the proportion that achieved lumefantrine concentrations ≥ 200 ng/mL between participants who had and did not have malaria recurrence by day 42.

In an exploratory analysis, none of the participant characteristics predicted risk of malaria recurrence neither did they predict risk of re-infection among those with available PCR results by day 42 of follow-up (see Additional file 3).

### Serious adverse events and grade 3 or 4 treatment emergent adverse events

From enrolment to follow-up day 63, there was one SAE and two cases of grade 3 neutropaenia. The SAE was lobar pneumonia, confirmed on chest examination and radiography, that occurred on day 14 of follow-up in a 31 years old female who had a baseline CD4 cell count of 164/μL and was not on cotrimoxazole prophylaxis. She was hospitalized and treated with intravenous antibiotics. The lobar pneumonia resolved by day 28 of follow-up without sequela. The cases of grade 3 neutropaenia were in two male participants, 36 and 34 years old, who had baseline neutropaenia of grade 2 that worsened to grade 3 by day 2 of treatment. In both cases, the neutropaenia resolved by day 7 of follow-up. None of these cases was judged to be probably related to intake of AL.

### QT interval abnormalities

At baseline, only 3.9% (6/152) of participants had predose QTcF ≥ 450 ms. A change in QTcF interval of > 60 ms from baseline to day 2 occurred in 17.1% (26/152) of the patients (Table 3). The observed QTcF interval abnormalities resolved by day 14 of follow-up. No cardiovascular abnormalities were detected in these individuals.

**Table 3:** Median Fridericia corrected QT interval (QTcF) and proportion with abnormal ECG findings from baseline to last day of dosing among participants on artemether–lumefantrine and efavirenz-based antiretroviral therapy in the intention-to-treat population (N = 152)

	Median (IQR) QTcF in msec	Proportion with QTcF ≥ 450 ms	Proportion with QTcF change > 60 ms from baseline to last day of dosing on day 2 (taken within 2 h of dosing)
Time of ECG test	NA	*n (%)*	*n (%)*
Day 0	393 (358–413)	6 (3.9)	NA
Day 1	392 (358–413)	6 (3.9)	19 (12.3)
Day 2	384 (359–402)	1 (0.7)	26 (17.1)
Follow-up visit after day 2 (day 7 or 14 of follow up)	392 (384–436)	0	NA

*IQR* interquartile range, *ECG* electrocardiogram

### Haematological parameters

The mean (SD) haemoglobin concentrations remained unchanged from baseline to day 7 (efavirenz-ART group: 11.3 to 11.2 g/dL (p = 0.592) but slightly increased from day 7 up to day 42, from 11.2 to 11.6 g/dL (p < 0.001). The median (IQR) CD4 cell count increased significantly from baseline to day 28, from 376 (248–511) to 458 (324–624) (p < 0.001).

## Discussion

The World Health Organization recommends the use of first-line anti-malarial drugs with cure rates of > 95% and changing anti-malarial drugs with cure rates of less than 90% [4]. This study was designed to assess whether AL achieves a day-42 PCR-adjusted malaria cure rate of 90% in HIV-infected adults on efavirenz-based ART with uncomplicated falciparum malaria. AL achieved a day-42 PCR-adjusted malaria cure rate of 100% in this sub-population living in an area of moderate-to-high malaria transmission in northern Zambia. Even in the worst case scenario, where participants with missing PCR results by day 42 were considered as treatment failures, the PCR-adjusted malaria cure rate was still > 94%. Additionally, AL achieved parasite clearance rates, similar to that in HIV-uninfected individuals [31–33]. Although there was no evidence of malaria recrudescence, at least 16% of the study participants had malaria recurrence by day 42, with the majority of the re-infections occurring between days 35 and 42.

A few studies have previously examined the efficacy of AL in HIV-infected non-pregnant adults on efavirenz-based ART. A Tanzanian study found that AL achieved a day-28 PCR-unadjusted cure rate of 82.5% in HIV-malaria co-infected patients on efavirenz-based ART [11]. In this study, which had longer follow-up period, the day-42 PCR-unadjusted cure rate was 83.6% while the day-28 cure rate was 98.0%. It is difficult to make direct comparisons of findings from the two studies because, compared with the Tanzanian study participants, participants in this study had a higher median CD4 cell count, lower median baseline parasite density and lower median temperature at the time of presentation. Nevertheless, similar to the Tanzanian study, this study did not find any cases of early treatment failure suggesting a high efficacy of AL in clearing malaria parasites.

Previous pharmacokinetic studies have found lower lumefantrine and artemisinin concentrations in individuals on efavirenz-based ART than in ART-naïve individuals [8–10]. Although the incidence of malaria recurrence was high in this study, significant differences in median lumefantrine concentration were found between individuals with and without malaria recurrence, or those with confirmed re-infections, suggesting that lumefantrine concentrations had minimal impact on malaria recurrence, including re-infections. Previously, day-7 lumefantrine concentrations of ≥ 200 ng/mL have been shown to be a surrogate marker of overall lumefantrine exposure and they predict AL treatment success by days 28 or 42 of follow-up [34], with concentrations below this level being associated with increased risk of malaria recurrence (both recrudescence and reinfections). This study found no significant difference in the proportion that achieved day-7 lumefantrine concentrations ≥ 200 ng/mL between participants who experienced malaria recurrence and those who did not experience malaria recurrence. These results contrast with those from a previous Tanzanian study [11] which found that malaria patients on ART who had recurrent parasitaemia by day 28, after treatment with AL, had lower median lumefantrine concentrations than those who remained aparasitaemic and that the proportion that achieved day-7 lumefantrine concentrations ≥ 280 ng/mL was higher in those who remained aparasitaemic. Reasons for the discrepancies are unclear. Nevertheless, participants in this study had a lower median day-7 lumefantrine concentration than the Tanzanian participants, suggesting that they were likely to have sub-therapeutic lumefantrine concentrations in the late post-treatment days 28–42 when most of the recurrent malaria infections occurred. The difference in day-7 lumefantrine concentrations in the two studies could be due to many factors, including genetic variations in CYP450 enzymes (CYP3A4 and CYP2B6) between the populations which metabolize ACT and efavirenz [35].

The finding of lack of evidence of an association between day-42 malaria recurrence and day-7 lumefantrine concentrations highlights that day-7 concentrations are unlikely to be predictive of malaria recurrence beyond 28 days following malaria illness. This is similar to what has been previously shown in a pooled analysis of data from HIV-uninfected individuals treated for uncomplicated falciparum malaria [34].

In this study, AL was very well tolerated, as observed in studies involving HIV-negative individuals [32, 36, 37]. Study participants also experienced marked improvement in CD4 count and haemoglobin levels following malaria treatment. Nevertheless, there were two cases of grade 3 neutropaenia that were judged as ‘unlikely related’ to AL. Also, 17.1% of the study participants had prolonged QTcF (> 60 ms) after AL treatment but none had an absoloute QTcF interval of > 500 ms or any clinically detectable cardiac events. It is possible that the prolonged QTcF, which resolved by day 14 of follow-up, may have been due to fever resolution [38, 39] rather than lumefantrine toxicity. Taken together, these observations confirm the safety of AL in HIV-infected individuals on efavirenz-based ART which is in agreement with previous findings [10, 11] and are in line with WHO’s recommendation on cardiac safety of ACT using the current standard treatment doses [40].

Understanding other factors, besides lumefantrine concentrations, associated with malaria recurrent malaria infections in HIV-co-infected individuals treated with AL can inform the optimal management strategies for malaria in this sub-population. This study did not find evidence of any significant association between patient characteristics, including use of cotrimoxazole prophylaxis or malaria parasite clearance rate, and malaria recurrence by day 42 (Additional file 3). In contrast, a previous pooled analysis of individual participant data from Asia and Africa found that baseline parasite density predicted risk of malaria recurrence among HIV-uninfected individuals [41]. Participants in this study had relatively low initial parasitaemias, experienced a rapid parasite clearance and there were no cases of early treatment failure which may explain the lack of association between baseline parasite density and malaria recurrence. Also, consistent with a previous Tanzanian study [11], this study found no significant association between baseline CD4 cell count and risk of malaria recurrence. Most of the study participants experienced improvements in CD4 counts from baseline to post-treatment days 28 and 42, hence, baseline CD4 count was not a good marker of status of immunity in the post-treatment period when people were susceptible to malaria re-infections. This study was not designed or powered to identify predictors of malaria re-infections but future studies should do so.

The strengths of this study were that AL treatment doses were administered under direct observation, drug tolerability was closely monitored in hospital and the loss to follow-up rate was low. One limitation of the study was the missing PCR results in the few patients that experienced malaria recurrence. However, the missing data are unlikely to have significantly affected accurate estimation of efficacy level of AL as highlighted in the sensitivity analyses. Another limitation was that the study cohort had a low median parasite density, hence the presence of non-malarial febrile illnesses cannot be ruled out. The high cure rates found in this study could have been due to the clearance of incidental low density parasitaemia which would have otherwise not required treatment. Nevertheless, AL still achieved rapid parasite clearance among a sub-set of participants with high parasite densities and also achieved rapid fever clearance in patients who were febrile at baseline but received no additional treatment for other febrile illnesses. Due to lack of pharmacogenetic data, this study did not investigate the impact of polymorphisms in CYP2B6 enzymes on plasma efavirenz concentrations which in turn can result in higher plasma efavirenz concentrations available to induce the enzymes that metabolize lumefantrine (CYP3A4). Future studies should aim to assess this impact.

## Conclusions

This study found that AL was safe and efficacious when used to treat uncomplicated falciparum malaria among HIV-infected adults on efavirenz-based ART which provides evidence for maintaining the same AL dosing regimens used in HIV-uninfected individuals with uncomplicated malaria. Nevertheless, a higher than expected proportion of participants with malaria re-infections was observed, which warrants the use of additional malaria prevention interventions in HIV-infected adults on efavirenz-based ART.

## Additional files

**Additional file 1.** Day-42 PCR-adjusted efficacy plot. PCR-adjusted ACPR by day 42 in the intention-to-treat population among malaria-HIV co-infected patients who were on efavirenz-based ART and were treated for malaria with artemether-lumefantrine. Participants with missing PCR results at day 42 (n = 8) are excluded from the plot.

**Additional file 2.** Day-42 PCR-unadjusted efficacy plot. PCR-adjusted ACPR by day 42 in the intention-to-treat population among malaria-HIV-co-infected patients who were on efavirenz-based ART and were treated for malaria with artemether-lumefantrine.

**Additional file 3.** Table on Cox regression analysis of risk of recrudescence by day 42 of follow-up.

## Data Availability

Data from this trial are held at the Malawi Liverpool Wellcome Trust Clinical Research Programme (MLW) which encourages optimal use of data by employing controlled access approach to data sharing with a robust system to review requests for data use and provide secure data access that is consistent with relevant ethics committee approvals. The datasets are therefore not publicly available but are available on reasonable request from the corresponding author and requests can also be initiated by contacting MLW: data@mlw.mw.

## Abbreviations

AE: adverse event
ACT: artemisinin-based combination therapy
ACPR: adequate clinical and parasitological response
ART: antiretroviral therapy
AL: artemether–lumefantrine
