# Supplementary figures and images for: Efficacy and safety of artemether–lumefantrine as treatment for Plasmodium falciparum uncomplicated malaria in adult patients on efavirenz-based antiretroviral therapy in Zambia: an open label non-randomized interventional trial
Source: Malar J. 2019 May 24;18:180. doi: 10.1186/s12936-019-2818-7 (PMC6534937; doi:10.1186/s12936-019-2818-7)

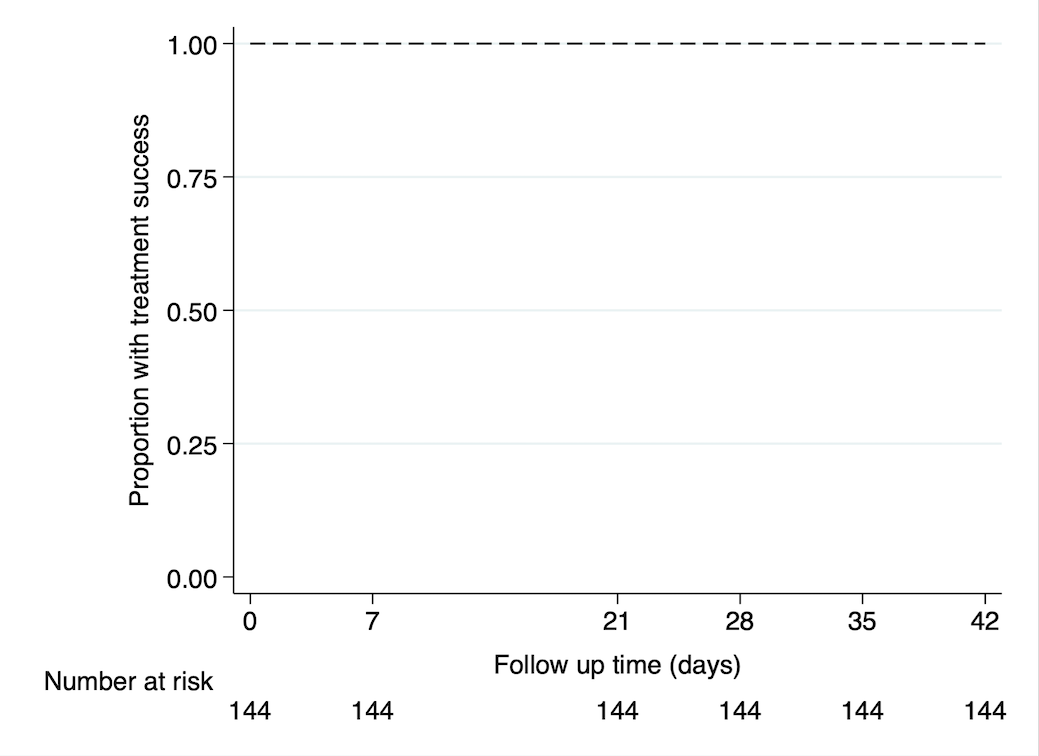

Supplement: Supplementary file 1 — Additional file 1. Day-42 PCR-adjusted efficacy plot. PCR-adjusted ACPR by day 42 in the intention-to-treat population among malaria-HIV co-infected patients who were on efavirenz-based ART and were treated for malaria with artemether-lumefantrine. Participants with missing PCR results at day 42 (n = 8) are excluded from the plot. [file 12936_2019_2818_MOESM1_ESM.tif]

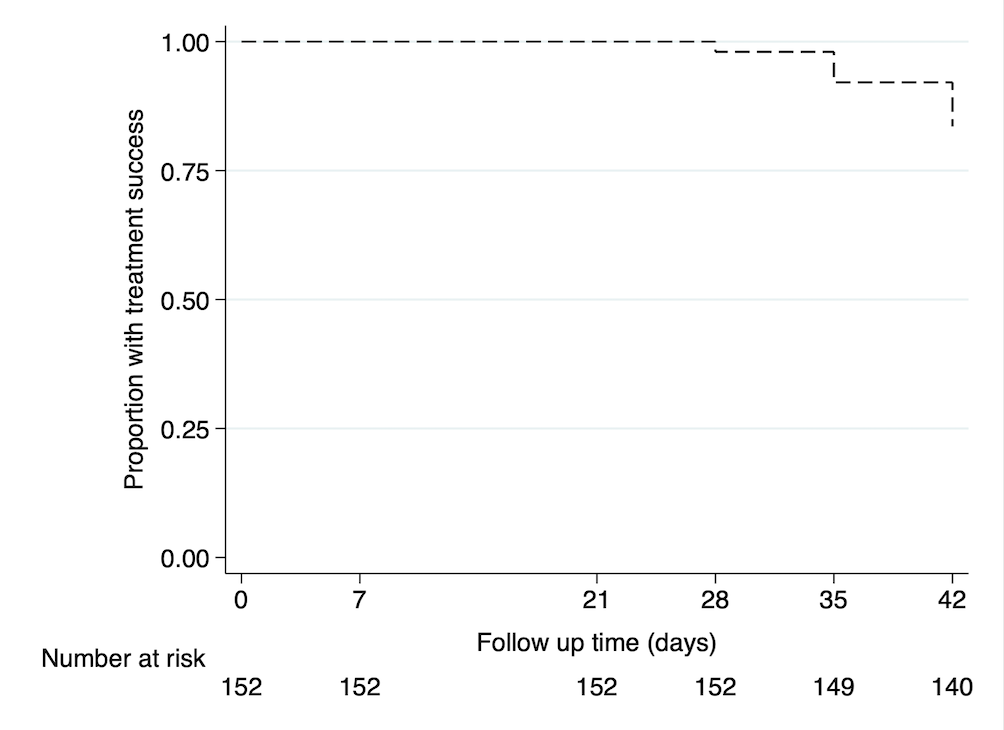

Supplement: Supplementary file 2 — Additional file 2. Day-42 PCR-unadjusted efficacy plot. PCR-adjusted ACPR by day 42 in the intention-to-treat population among malaria-HIV-co-infected patients who were on efavirenz-based ART and were treated for malaria with artemether-lumefantrine. [file 12936_2019_2818_MOESM2_ESM.tif]
