# Supplementary material for: Efficacy and safety of artemether–lumefantrine as treatment for Plasmodium falciparum uncomplicated malaria in adult patients on efavirenz-based antiretroviral therapy in Zambia: an open label non-randomized interventional trial
Source: Malar J. 2019 May 24;18:180. doi: 10.1186/s12936-019-2818-7 (PMC6534937; doi:10.1186/s12936-019-2818-7)
Supplement: Supplementary file 3 — Additional file 3. Table on Cox regression analysis of risk of recrudescence by day 42 of follow-up. [file 12936_2019_2818_MOESM3_ESM.docx]

| **Additional file 3: Table on Cox regression analysis of risk of malaria recurrence by day 42** | | | | | | | |  | |  | |  | |  | |  | |  | |
| --- | --- | --- | --- | --- | --- | --- | --- | --- | --- | --- | --- | --- | --- | --- | --- | --- | --- | --- | --- |
| **Variable** | | **Number of patients** | | **Number of events (malaria recurrence)** | **Hazard Ratio**  **(95% CI)** | **P-value** | |  | |  | |  | |  | |  | |  | |
| *Univariate model *** |  |  | |  | |  | |  | |  | |  | |  | |  | |  | |
| Age in years | 152 | 25 | | 1.01 (0.98-1.05) | | 0.508 | |  | |  | |  | |  | |  | |  | |
| Sex (Female) | 101 | 17 | | 1.15 (0.43-2.32) | | 0.999 | |  | |  | |  | |  | |  | |  | |
| ART duration (in months) | 147 | 24 | | 1.00 (0.98-1.02) | | 0.629 | |  | |  | |  | |  | |  | |  | |
| Baseline CD4 cell count/mL | 151 | 25 | | 1.00 (0.99-1.00) | | 0.303 | |  | |  | |  | |  | |  | |  | |
| <350 | 67 | 9 | | 1.29 (0.46-3.62) | | 0.631 | |  | |  | |  | |  | |  | |  | |
| Not using cotrimoxazole prophylaxis | 95 | 18 | | 1.76 (0.70-4.43) | | 0.584 | |  | |  | |  | |  | |  | |  | |
| Baseline temperature | 152 | 25 | | 0.80 (0.40-1.60) | | 0.931 | |  | |  | |  | |  | |  | |  | |
| Fever (≥37.5℃) | 14 | 2 | | 0.81 (0.19-3.44) | | 0.521 | |  | |  | |  | |  | |  | |  | |
| Baseline haemoglobin in g/dL | 152 | 25 | | 1.06 (0.83-1.37) | | 0.602 | |  | |  | |  | |  | |  | |  | |
| Weight in Kgs | 144 | 17 | | 0.98 (0.92-1.04) | | 0.549 | |  | |  | |  | |  | |  | |  | |
| BMI |  |  | |  | |  | |  | |  | |  | |  | |  | |  | |
| Normal (18.5-24.9) | 99 | 15 | | Reference | | - | |  | |  | |  | |  | |  | |  | |
| Underweight (<18.5) | 45 | 10 | | 1.52 (0.68-3.38) | | 0.305 | |  | |  | |  | |  | |  | |  | |
| Overweight/Obese (>25.0) | 8 | 0 | | - | | - | |  | |  | |  | |  | |  | |  | |
| Baseline parasite count (log _10_) | 152 | 17 | | 0.60 (0.34-1.06) | | 0.078 | |  | |  | |  | |  | |  | |  | |
| >2000 parasites/µL | 53 | 8 | | 0.83 (0.36-1.91) | | 0.655 | |  | |  | |  | |  | |  | |  | |
| >10 000 parasites/µL | 19 | 2 | | 0.57 (0.14-2.44) | | 0.452 | |  | |  | |  | |  | |  | |  | |
| Parasite clearance half-life (in hours) | 57 | 8 | | 0.76 (0.37-1.53) | | 0.441 | |  | |  | |  | |  | |  | |  | |
| Daily mg/kg dose of lumefantrine received | 152 | 25 | | 1.01 (0.88-1.16) | | 0.889 | |  | |  | |  | |  | |  | |  | |
| Day 7 lumefantrine concentration (log _10_) | 85 | 13 | | 0.62 (0.11-3.43) | | 0.591 | |  | |  | |  | |  | |  | |  | |
| <200 ng/ml | 34 | 4 | | 0.64 (0.20-2.08) | | 0.460 | |  | |  | |  | |  | |  | |  | |
| **None of the determinants in the univariate model showed significant association with malaria recurrence by day  42 (P-value <0.05). Therefore, multivariate Cox regression model not fitted | | | | | | | | | | | | | | | | | | | |
| ** Sensitivity analysis, excluding 8 participants with missing PCR samples (n=144) did not result in significant association  of any covariates with risk of malaria reinfection by day 42 | | | | | | | | | | | | | | | | | |  | |
